# EXPRESSION OF CONCERN: Subtype‐Specific Alterations of the Wnt Signaling Pathway in Breast Cancer: Clinical and Prognostic Significance

**DOI:** 10.1111/cas.16460

**Published:** 2025-01-24

## Abstract

**EXPRESSION OF CONCERN:** N. Mukherjee, N. Bhattacharya, N. Alam, A. Roy, S. Roychoudhury, and C.K. Panda, “Subtype‐Specific Alterations of the Wnt Signaling Pathway in Breast Cancer: Clinical and Prognostic Significance,” *Cancer Science* 103, no. 2 (2012): 210–220, https://doi.org/10.1111/j.1349‐7006.2011.02131.x.

This Expression of Concern is for the above article, published online on 25 October 2011, in Wiley Online Library (wileyonlinelibrary.com), and has been issued by agreement between the journal Editor‐in‐Chief, Masanori Hatakeyama; the Japanese Cancer Association; and John Wiley & Sons Australia Ltd. The Expression of Concern has been agreed due to concerns raised by a third party after publication regarding the similarity of certain bands in Figure 2b and the underlying data that they represent. The authors could not provide the original data given the time that had elapsed since publication. Because the publisher was not able to examine the original data, it was not possible to investigate fully the concerns raised. Therefore, the journal is issuing this Expression of Concern because the concerns regarding the integrity of the data and the results presented cannot be resolved. Chinmay K. Panda agrees with this decision on behalf of the authors.

